# Genome-Wide Identification of Potential mRNAs in Drought Response in Wheat (*Triticum aestivum* L.)

**DOI:** 10.3390/genes13101906

**Published:** 2022-10-20

**Authors:** Muhammad Aqeel, Wajya Ajmal, Quratulain Mujahid, Maryam Murtaza, Mansour Almuqbil, Shakira Ghazanfar, Muhammad Uzair, Ayesha Wadood, Syed Mohammed Basheeruddin Asdaq, Rameesha Abid, Ghulam Muhammad Ali, Muhammad Ramzan Khan

**Affiliations:** 1 National Institute for Genomics and Advanced Biotechnology, National Agricultural Research Centre, Park Road, Islamabad 45500, Pakistan; 2 Department of Botany, Lahore College for Women University Lahore, Lahore 54000, Pakistan; 3 Department of Clinical Pharmacy, College of Pharmacy, King Saud University, Riyadh 11451, Saudi Arabia; 4 National Key Facility for Crop Gene Resources and Genetic Improvement, Institute of Crop Sciences, Chinese Academy of Agricultural Sciences, Beijing 100081, China; 5 Department of Biochemistry, Quaid-i-Azam University, Islamabad 45320, Pakistan; 6 Department of Pharmacy Practice, College of Pharmacy, AlMaarefa University, Dariyah, Riyadh 13713, Saudi Arabia or; 7 Pakistan Agricultural Research Council, Islamabad 44000, Pakistan

**Keywords:** drought, bread wheat, meta data, RNA seq, genomics

## Abstract

Plant cell metabolism inevitably forms an important drought-responsive mechanism, which halts crop productivity. Globally, more than 30% of the total harvested area was affected by dehydration. RNA-seq technology has enabled biologists to identify stress-responsive genes in relatively quick times. However, one shortcoming of this technology is the inconsistent data generation compared to other parts of the world. So, we have tried, here, to generate a consensus by analyzing meta-transcriptomic data available in the public microarray database GEO NCBI. In this way, the aim was set, here, to identify stress genes commonly identified as differentially expressed (*p* < 0.05) then followed by downstream analyses. The search term “Drought in wheat” resulted in 233 microarray experiments from the GEO NCBI database. After discarding empty datasets containing no expression data, the large-scale meta-transcriptome analytics and one sample proportional test were carried out (Bonferroni adjusted *p* < 0.05) to reveal a set of 11 drought-responsive genes on a global scale. The annotation of these genes revealed that the transcription factor activity of RNA polymerase II and sequence-specific DNA-binding mechanism had a significant role during the drought response in wheat. Similarly, the primary root differentiation zone annotations, controlled by *TraesCS5A02G456300* and *TraesCS7B02G243600* genes, were found as top-enriched terms (*p* < 0.05 and Q < 0.05). The resultant standard drought genes, glycosyltransferase; Arabidopsis thaliana KNOTTED-like; bHLH family protein; Probable helicase MAGATAMA 3; SBP family protein; Cytochrome c oxidase subunit 2; Trihelix family protein; Mic1 domain-containing protein; ERF family protein; HD-ZIP I protein; and ERF family protein, are important in terms of their worldwide proved link with stress. From a future perspective, this study could be important in a breeding program contributing to increased crop yield. Moreover, the wheat varieties could be identified as drought-resistant/sensitive based on the nature of gene expression levels.

## 1. Introduction

More than two billion people worldwide, particularly in Asia, depend on wheat (*Triticum aestivum* L.) as their primary source of proteins, B vitamins, and dietary fibers [1,2]. Wheat is the largest produced commodity, reaching, approximately, 778.6 million metric tons [3]. A record 194 million tons of wheat will be traded globally in 2022, up 2.5 percent (4.8 million tons) from the level in 2020/21. As a result of a better-than-anticipated demand, this represents a rise of 1.1 million tons from the previous report [4]. It is also predicted that in order to meet the demand and consumption in 2050, wheat production will need to rise by 50–60% from its current level.

Wheat biomass and yield are reduced by 25.0% and 27.5%, respectively, by drought [5]. To meet the problems of ensuring nutritional security, wheat grain quality must also increase in terms of its ability to withstand drought [6]. The frequency of rainfall, evaporation, and soil moisture all play a role in how severe or intense drought is, among other factors [7]. Drought stress not only affects more than one-third of all farmed land worldwide, but also facilitates insect infestation leading to low crop productivity. A total of 33% (9.9 × 10^7^ hm^2^) of that territory is occupied by developing nations, 25% (6.0 × 10^7^ hm^2^) by developed countries, and 42% (12.6 × 10^7^ hm^2^) by underdeveloped states [8]. In Asia, there is only 3.4 × 10^7^ hm^2^ of rainfed lowland and 8.0 × 10^6^ hm^2^ of highland wheat that is under drought stress [9]. Wheat productivity can be increased in a sustainable and economically feasible way by breeding wheat types that are tolerant to drought stress [10]. Previous research has shown that drought has a negative influence on crop production. Given the inherent problems with drought stress for wheat breeders, a detailed understanding of the molecular basis of wheat drought stress can assist greatly in the identification of appropriate varieties. Numerous genes and microbial interactions are activated as a result of the drought-stress response in plants [1,11,12,13,14]. The wheat *NAC* gene (*TaNAC071-A*) is recently reported as showing one of the strongest associations with drought stress. They experimented with the knockout technique and found that the *TaNAC071-A* gene is a positive regulator of plant dehydration response [15,16]; Zinc finger protein (*ZFP*) consists of various member proteins constituting a family [17], apart from the RNA/DNA difference (RDD) during central dogma confer resistance to drought stress in plants [18].

The genes are included in selenium stress [19], response to auxin [20], electron transport chain, transcription regulated by the RNA polymerase II promoter, and different transcription factors. The primary transcription factors associated with stress include *TraesCS3D02G120200*, *TraesCS6A02G328700*, *Traes-CS2D02G000200*, *TraesCS6D02G086600*, *TraesCS6D02G260700*, *TraesCSU02G154600*, *TraesCS6B02G234100*, *TraesCS1D02G333000*, *TraesCS5A02G456300*, *TraesCS6A02G24-0400*, and *TraesCS7B02G243600;* these genes are crucial for controlling drought tolerance [21,22,23,24].

This research included a number of meta-analyses, summary studies, and model simulation results that only took drought into consideration, and discarded samples that had been subjected to stress other than drought. Under the conditions of climate- and water-availability constraints, it will be important to boost biomass production and economic yield. Before that can happen, it is necessary to comprehend the magnitude of the decline in wheat production and other agronomic features that are impacted by molecular parameters, such as drought proteins. Apart from generating knowledge of global drought genes, we are also interested to conduct an assessment of our methodology as a strategy of metatranscriptome analytics scale to address drought stress. In the future, this standardized methodology can serve as a road map of the potential for application in other crop species.

## 2. Materials and Methods

### 2.1. Meta-Analysis of Data from the Genome-Wide Transcriptome

**Figure 1:**
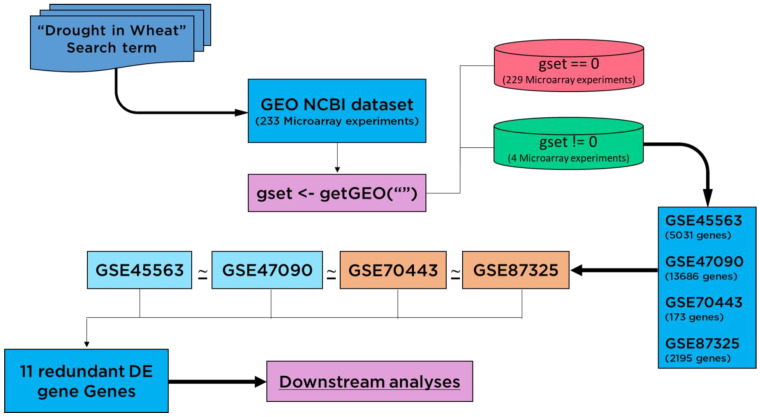
Flow chart diagram shown for screening of differentially expressed (DE) genes. Redundant genes were collected using expression datasets which were differentially expressed in each dataset.

The GEO Datasets [25] GSE45563 (without heat stress samples), GSE47090, GSE70443, and GSE87325 were employed as data sources. The expression profiles of drought-related genes (Figure 1) were targeted for *Triticum aestivum,* obtained in control and stress (water shortage) conditions [26]. The search term “Drought in wheat” was used to search for 233 microarray experiments from the GEO NCBI database. To identify changes in the expression patterns of genes, the expression profiles were downloaded using the *getGEO()* function. Out of 233 datasets, the empty experiments were discarded, containing no expression data by using the condition *gset* = 0. The large-scale meta-transcriptome analytics and 1 sample proportional test (Bonferroni adjusted *p* < 0.05) revealed a set of 11 drought-responsive genes on a global scale.

### 2.2. Differential Expression Analyses

Researchers in bioinformatics have gained a lot of knowledge from studying microarray data. For instance, information sharing across all probes can increase the power to detect differential expressions and decrease false findings. One such method is limma [27], where the probe-wise variances were moderated using an empirical Bayes model. In the t- and F-statistic calculations, the moderated variances take the place of the probe-wise variances. EdgeR models count data using an over-dispersed Poisson model in a conceptually similar, but theoretically more challenging processes, and uses an empirical Bayes approach to control the degree of overdispersion among genes.

We anticipated that the information could be condensed into a table of counts, with rows denoting genes (or tags, exons, or transcripts) and columns denoting samples. These could be counts at the exon, transcript, or gene levels for RNA-seq research. The data are modelled using a negative binomial (NB) distribution,

(1) $$Y_{ki}\sim NB\left(M_{i}p_{ki},\phi_{k}\right)$$

for sample *i* and gene *g*. Here, *Mi* is the size of the library (the total number of reads), *ϕ_k_*. denotes the dispersion, and *pkj* denotes the relative abundance of the gene *g* in experimental group *j*, which includes sample *i*. We use the *NB* parameterization where the mean is *μ_ki_* = *M_i_p_kj_* and the variance is *μ_ki_* (1 + *μ_ki_ϕ_k_*). For differential expression analysis, the parameters of interest are *p_kj_*. The NB distribution was reduced to Poisson when *ϕ_k_* = 0. In some DGE applications, technical variation can be treated as Poisson. In general, *ϕ_k_* indicates the biological variation’s coefficient of variance between the samples. Our approach is able to distinguish between biological and technical variance in this manner.

EdgeR calculates the gene-wise dispersion probabilities using conditional maximum likelihood, based on the total number of genes in that gene [28]. The dispersions were reduced using an empirical Bayes approach to a consensus value, efficiently utilizing information from other genes. Finally, the differential expression for each gene was evaluated using an exact test similar to Fisher’s exact test but modified for highly distributed data.

R software (version 3.4.3; https://cran.r-project.org/) (accessed on 10 October 2022) was used to process drought-related microarray data acquired from the GEO database. The edgeR tool in R was used to identify differentially expressed genes (DEGs) across drought-stressed plants and control plants. Fold-change (FC) values were determined, and the following cutoff criteria were used to further select the DEGs: *p* < 0.05, FDR < 0.05 and log |FC| > 2. The four datasets’ overlapping DEGs were found using MS EXCEL manipulations (version 3.1.3; http://www.funrich.org) (accessed on 10 October 2022).

### 2.3. One-Sample Proportions Test for SDGs Screening

Only groups with finite numbers of gene presences and absences were used. The counts of presences and absences must be non-negative and, hence, not greater than the corresponding numbers of microarray experiments, which must be positive. All finite counts should be integers. In R, the prop.test function was utilized for >1 gene presences in groups. Thus, for 2, 3, and 4 presences, the proportions 50 by 100, 75 by 100, and 100 by 100 were used, respectively. Later on, 2 presences in microarray experiments with the proportion 50 by 100 were discarded due to *p* = 1 outcomes. The reproducibility can be checked using the R commands below:

> prop.test(50,100)$*p*.value

[1] 1

> prop.test(75,100)$*p*.value

[1] 9.583666e-07

> prop.test(100,100)$*p*.value

[1] 4.16275e-23

### 2.4. Bioinformatics Analyses

Sequence analysis and annotation predications were performed using the NCBI database containing BLAST tool. The default alignment parameter settings were as, max target sequences were restricted up to 100; threshold 100; Word size 28; Max matches in a query range 0; Match/Mismatch Scores 1, −2; Gap Costs were selected as linear; low-complexity region; mask for lookup table only during the BLAST search. Accession numbers against GI identifiers were identified, and then drought genes nucleotide and protein sequences were retrieved. PlantRegMap is a plant genomes database with advanced biocomputing tools (http://plantregmap.gao-lab.org/) (accessed on 10 October 2022). For transcription factor analysis, the protein sequences were aligned using the BLAST online tool in PlantTFDB v5.0 to mine for stress-related orthologs present in the database in the annotated form.

### 2.5. Functional and Pathway Enrichment Analyses of SDGs

An important resource for any functional investigation of the high-throughput gene-expression profiles is clusterProfiler v4.0.5, which we used for the functional annotation of GO and analysis of KEGG pathway enrichment [29]. The results from clusterProfiler were further imported to multienrichjam v17.900 in R v 4.1.1. The IGRAPH and TKPLOT were employed to illustrate the top 11 SDGs’ functional enlargement. To thoroughly examine the SDGs connected to the GO terms and pathways, we conducted an integrative analysis using both the clusterProfiler v4.0.5 and IGRAPH packages. To obtain all Gene Ontological terms (GO) and trait-related pathways from the SDG dataset, the initial SDGs from the GEO2R tool were exposed to clusterProfiler v2.5.5/multienrichjam v17.900. clusterProfiler syndicates GO from the SDG dataset, delivering a fundamentally ordered functional network. Additionally, the enrichment of molecular/biological function GO analysis for SDGs was carried out, and *p*-values 0.05 were deemed significant.

### 2.6. Principal Component Analyses

Principal components analysis of the SDGs and the own gene expression dataset were calculated in R version 4.1.1 using the PCA function of the FactoMineR v2.4 package. Subsequently, we compared the first and second PCs of both datasets in order to determine whether they span similar spaces.

### 2.7. Plant Ontology

A controlled vocabulary (ontology), known as Plant Ontology, explains the anatomy, morphology, and developmental stages of all plants. In order to enable meaningful cross-species queries on gene expression and phenotype data sets from plant genomics and genetics investigations, the PO aims to create a semantic framework.

The need to create new and better-adapted wheat varieties is brought on by the rise of the world’s population and the changing climate. The Plant Trait Ontology (TO), the Crop Ontology (CO), and the GrainGenes database are three cohesive resources that give scientists and plant breeders connected resources and tools to make use of the vast amounts of genetic and genomic data that are available for plant genomics and crop development. The Planteome Project’s reference-level ontology for plant characteristics is called the TO. More than 1500 plant traits are included in the TO of the most recent Planteome Release Version 4.0, which are arranged into nine higher-level categories: biochemical, biological, and plant growth and development processes, as well as plant shape, quality, vigour, stress, and yield. The TO is integrated with the Plant Ontology as part of the Planteome and is used to annotate or link to data objects for plant genomics and genetics (such as germplasm, QTLs, genes, and proteins) from a variety of plant taxa, including significant world crops and model plant species. There are around 165,000 data elements in the Planteome database that are linked by more than four million annotations in this release. Through the TO GitHub Issue Tracker, users are encouraged to request new TO terms or to leave comments.

## 3. Results

### 3.1. Meta-Analysis of Data from the Genome-Wide Transcriptome

For bread wheat *(T. aestivum),* the GEO Datasets [25] were used as a source of data for the expression profiles of drought-related genes acquired in control and stress (normal water and water deficit) situations [26]. Initially, the samples consisted of 233 experiments for wheat, out of which four experiments were screened for available transcriptome data (GSE87325, GSE47090, GSE45563 and GSE70443), including information from microarray and NGS platforms (Supplementary Table S1). Other drought-stressed samples were excluded from the analyses, such as GSE45563 which had heat and analyzing 233 RNAseq IDs. Of the datasets GSE87325, GSE47090, GSE45563 and GSE70443, two originated from the USA, one dataset was from Germany and one from India, and were acquired from the GEO NCBI database. All datasets were checked for expression availability, whether the link “Analyze with GEO2R” is provided or not. Luckily, we got four out of 233 of which the expression data was available in GEO NCBI.

### 3.2. Global Drought Genes Screening

**Figure 2:**
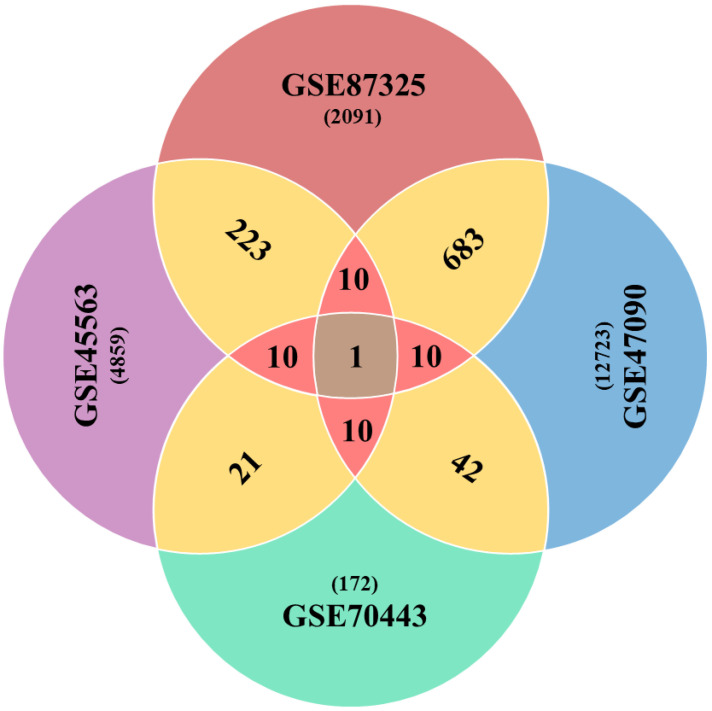
Venn diagram elaborating shared number of genes across the combinations of microarray datasets. Sharing of DE genes between two datasets are shown in gold color, while sharp pink color represents three combination outcomes, in terms of sharing the number of DE genes.

The differential expression analyses resulted in a variable number of genes in each case of the microarray experiment. These were 4859, 12,723, 172 and 2091 drought-related genes (*p* < 0.05) from GSE45563, GSE47090, GSE70443 and GSE87325 microarray experiments, respectively. After a series of analytics, we came up with one gene with the GI accession 31369563 as a global factor towards drought stress in wheat as it was found as differentially expressed among all microarray experiments. Another ten genes passed the one sample proportion test (*p* < 0.05) (Table 1). In total, we got a list of eleven Standard Drought Genes (SDGs). The pairwise comparison showed a larger number of DE genes, as shown in Figure 2. However, a comparison between three and four datasets was 10 and 1 DE genes for each combination, respectively.

### 3.3. IWGSC Gene IDs Identification

Annotating genes is core to every biological experiment. We ran the BLAST search using ENSEMBL database, against wheat as the reference genome. The top hits accessed from aligned results are shown in Table 2. The high E-value, along with the alignment score and identity percentage for top hits homologs, elucidated that they had a strong reason to be considered as orthologs of the drought genes.

### 3.4. Identification of Analyzed Genes Transcripts

This study analyzed genes encoding, glycosyltransferase, Auxin-responsive family protein, NAD(P)H-dependent oxidoreductase 1 (drought-responsive), proteins involved in gene expression regulations and auxin response. The EnsemblPlants *(*https://plants.ensembl.org) (accessed on 10 October 2022) database was used to find full-length cDNA sequences in the wheat genome (*Triticum aestivum* v. 2.2) (Table 2) 91 for genes producing antioxidant enzymes and enzymes involved in proline production. For identified sequences, Basic Local Alignment Search Tool (BLAST) for alignment resulted in accession IDs of the drought genes. It can be seen in the chromosome column that the D genome is at the top to have a larger number of drought genes, five out of eleven, *TraesCS3D02G120200*, *TraesCS2D02G000200*, *TraesCS6D02G086600*, *TraesCS6D02G260700* and *TraesCS1D02G333000* compared to any other chromosome. Genome A was depicted as second having SDGs the most after the D genome.

### 3.5. Mapping of SDGs on Wheat Genome

**Figure 3:**
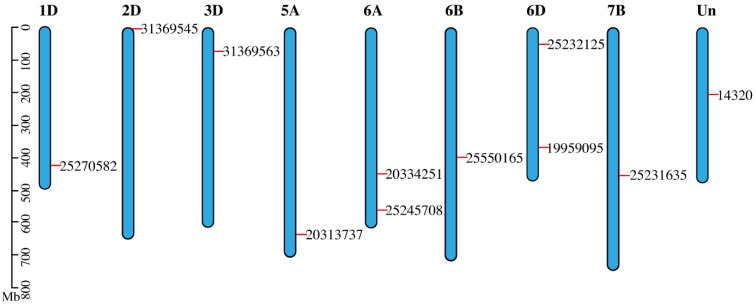
Chromosomal map depicted the SDGs distribution over their respective chromosomes. Out of all wheat chromosomes, all SDG-bearing chromosomes are included in the map. Gene places on chromosomes were made set according to their genomic locations. The chromosomes were drawn as blue bars.

Chromosomal map explained how to alter new tracks by visualizing chromatin state transit—chromatin state in the genome has moved, for example, from one group of samples to the other. Variable methylation patterns are present in the genomic areas where chromatin states change, and these patterns may be an intriguing indicator of how chromatin states change. Most gar chromosomes have two counterparts in sterlet, according to an examination of conserved syntenies between sterlet and gar. A total of 46 scaffolds were identified when sterlet homologous gene pairs were mapped against the genome in a paired manner. As expected from a WGD event, this finding shows homologous chromosomal fragments (Figure 3).

### 3.6. Expression Analyses

**Figure 4:**
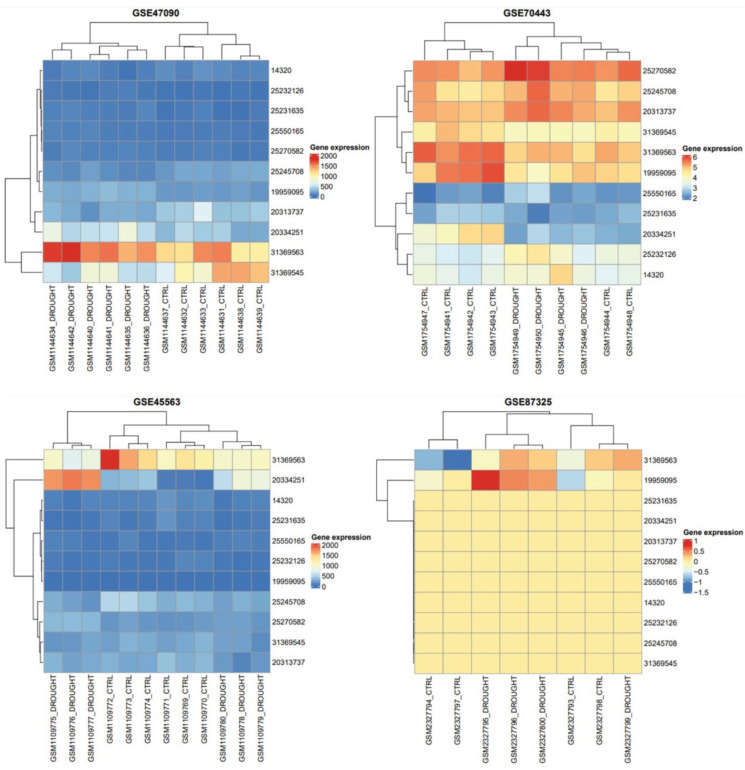
Heatmap and hierarchical clustering for gene expression matrices under well-watered and drought-stress conditions in 11 SDGs are shown as heatmaps. Color ranges are set between blue and red in order to visualize low to high gene expression, respectively. Clustering analysis of GSE47090, GSE70443, GSE45563 and GSE87325 showed two main horizontal groups of drought and controlled samples. Whereas, vertical scale is used to show all SDGs common in all gene expression datasets. Drought gene with GI accession 25550165 tends to be underexpressed in all experiments. Similarly, 313690563 gene in drought conditions had a trend towards overexpression in all gene expression datasets.

An understanding of a gene’s function in numerous biological processes can be gained by studying its expression profile. The expression studies of *T. aestivum* mRNAs were compared to drought and control phases in order to uncover the role of mRNA in distinct tissue developmental stages. Similar to the mRNAs, the majority of mRNA demonstrated differential expressions between the stages of drought and control (Figure 4). The results depicted their role in unrelated stages.

Physiological measures were utilized to generate a heatmap in order to pinpoint the critical variables for evaluating drought tolerance in wheat. As shown in Figure 2, for hierarchical (row) clustering, the morphological and physiological parameters of the 49 genotypes, which were grown either with drought treatment or with adequate water, were used. The 49 genotypes grouped together into group A when cultivated in well-watered conditions, while the identical 49 genotypes grouped together into group B when grown in drought-stricken conditions. By clear clustering, it can be seen that each switchgrass genotype’s physiological and morphological traits are changed by drought-stress treatment in comparison to control circumstances. Surprisingly, in well-watered areas, the majority of the lowland genotypes tended to group together (group a in Figure 2, dot-highlighted); however, under drought-stress conditions, these genotypes were dispersed (group b in Figure 2 dot-highlighted).

A heatmap for SDGs is drawn from their expression pattern, observed in all microarray datasets. Heatmap of RNA-Seq transcriptome analysis for 11 chosen genes from the Triticum aestivum complex drought group and reference IWGSC genome. On the basis of a number of analysis parameters, about 11 genes are displayed. Based on average linkage and Euclidean distance of gene expression data, genes and samples were hierarchically clustered (dendrogram is shown for genes). It can be seen that drought and control samples showed variation in their protein secretion when we applied different watering conditions. The gene expression of each drought gene in terms of box colors provided the idea about whether it is under- or over-represented (Figure 4).

### 3.7. Statistical Analysis

**Figure 5:**
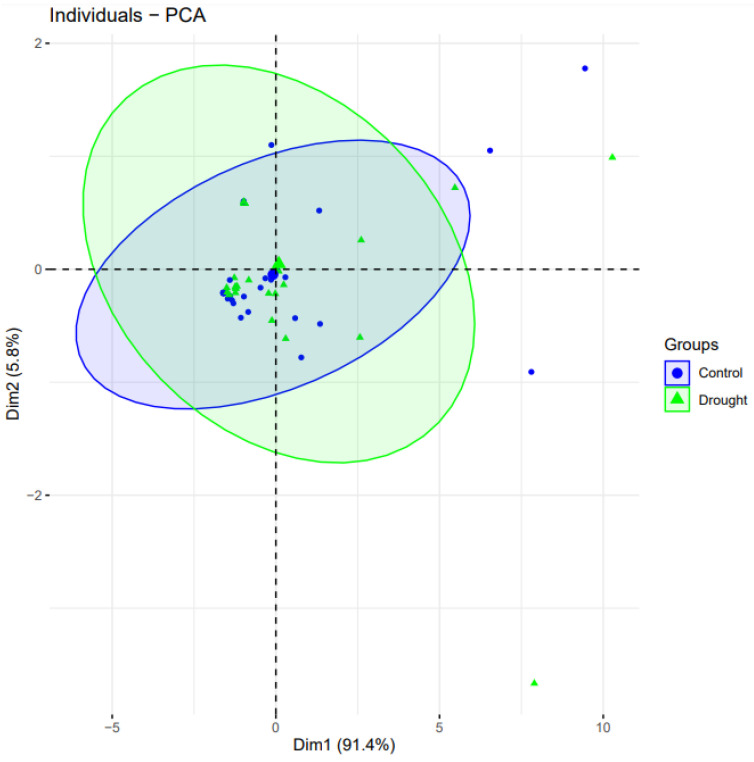
Principal component analysis biplot of gene expression of 11 genes studied in well-watered and drought conditions. PCA plot reveals the variation in the form of principal components (PC) 1 and 2. PC1 and PC2 drawn as Dim1 and Dim2 on horizontal and vertical axis, respectively, accounting for the variation up to 91.4% and 5.8%, respectively. Same genes are placed in plot based on their expression in drought and control conditions.

Clustering and principal component analysis (PCA) was obtained using the “factoextra” program [30]. We used PCA to assess each drought gene’s contributions to the drought-treated and control wheat plants. The genes from the control case were more important in separating the groups than the parameters that had been subjected to drought. (Figure 5). Among the eleven drought genes, three genes from control condition samples (blue-colored data points) covered an overall greater distance compared to the drought-treated groups, and thus, are considered positively correlated.

### 3.8. GO Categorization of SDGs

**Figure 6:**
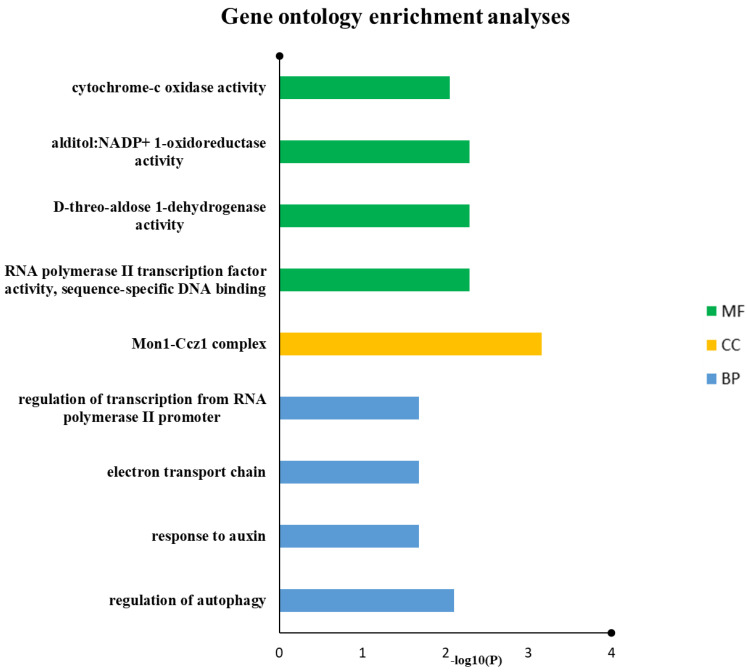
Drought proteins were annotated using reference genome of wheat from IWGSC. Predicted biological mechanisms indicated that auxin-related response, autophagy, electron transport chain and transcription regulations are prominent in this study.

GO keywords were given to 10 out of 11 drought genes, according to the gene ontology analysis. In biological process classification, the drought genes were involved in the regulation of autophagy, response to auxin, electron transport chain and regulation of transcription from RNA polymerase II promoter. In molecular function categorization, RNA polymerase II transcription factor activity, sequence-specific DNA binding, D-threo-aldose 1-dehydrogenase activity, alditol: NADP+ 1-oxidoreductase activity and cytochrome-c oxidase activity were the most popular terms, especially in the field of cellular components, and Mon1-Ccz1 complex was the most dominant terms (Figure 6 and Table 3).

### 3.9. Gene Annotation from PlantRegMap Database

The putative restrictive transcription factor was then found using our SDGs. We assumed that some of the linked genes may have known activities in the fruit dehiscence zone. To test this, we analyzed the smaller 11 gene set by first mapping each of the transcripts to the *Arabidopsis thaliana*, *Dichanthelium oligosanthes*, *Klebsormidium flaccidum*, *Medicago truncatula*, *Trifolium pratense*, *Utricularia gibba*, *Zea mays* and *Zoysia matrella* proteome, in order to find homologous gene loci, and those with E-values under 0.05 were chosen. Ten transcripts in all met this requirement, as shown in Table 4, and were then subjected to proxy analysis, using the most compatible *Arabidopsis thaliana*, *Dichanthelium oligosanthes*, *Klebsormidium flaccidum*, *Medicago truncatula*, *Trifolium pratense*, *Utricularia gibba*, *Zea mays* and *Zoysia matrella* gene locus IDs to conduct Plant Ontology (PO) and network analysis using PlantRegMap, to enable inference of putative gene function and regulatory networks. According to Table 5 linked PO keywords, 10 out of 11 drought gene homologues were found in the PlantRegMap database.

### 3.10. Plant Ontology Annotation Using Annotated Reference Genomes

**Figure 7:**
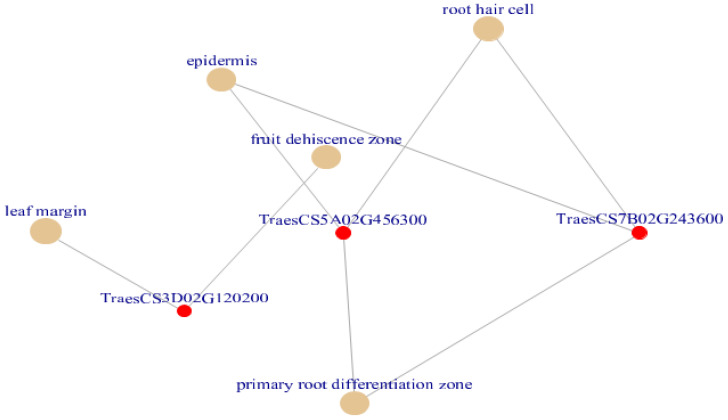
Cnetplot created using plant ontology enriched terms (Adjusted *p* < 0.05). Out of all enriched terms epidermis, root hair cell, lead margin, fruit dehiscence zone and primary root differentiation zone terms are plotted by default which was associated with drought in wheat. The terms primary root differentiation zone, epidermis and root hair are governed by the DE genes are TraesCS5A02G456300 and TraesCS7B02G243600.

The gene collection was collectively annotated for plant keywords, some of which we thought were compatible with BP-response to auxin (e.g., as for *TraesCS6A02G328700* with PO- cauline leaf, shoot apex, rib zone, inflorescence meristem, root, seed and plant embryo, vascular leaf, flower, sepal, petal, flower pedicel, gynoecium, receptacle, hypocotyl, collective leaf structure, plant embryo cotyledonary stage, mature plant embryo stage, plant embryo globular stage, plant embryo bilateral stage, petal differentiation and expansion stage and flowering stage); CC-cytosol; MF-oxidoreductase activity (e.g., as for *TraesCS2D02G000200* with PO-cauline leaf, shoot apex, inflorescence meristem, guard cell, leaf lamina base, shoot system, plant embryo, vascular leaf, stamen, carpel, sepal, petal, flower, stem, cotyledon, petiole, hypocotyl, leaf apex, collective leaf structure, vascular leaf senescent stage, plant embryo bilateral stage, LP.12 twelve leaves visible stage, LP.08 eight leaves visible stage, LP.02 two leaves visible stage, LP.10 ten leaves visible stage, LP.04 four leaves visible stage, LP.06 six leaves visible stage, petal differentiation and expansion stage, and flowering stage); CC-integral component of membrane (e.g., as for *TraesCS5A02G456300* with PO- root hair cell, guard cell, primary root differentiation zone, and epidermis) BP-regulation of transcription, DNA-templated; CC-nucleus; MF-DNA-binding transcription factor activity, RNA polymerase II-specific (e.g., as for *TraesCS6A02G240400* with PO- cauline leaf, shoot apex, inflorescence meristem, guard cell, leaf lamina base, fruit, root, shoot system, plant embryo, portion of vascular tissue, vascular leaf, carpel, petal, stamen, flower, sepal, stem, flower pedicel, cotyledon, hypocotyl, collective leaf structure, pollen, vascular leaf senescent stage, plant embryo globular stage, LP.04 four leaves visible stage, LP.06 six leaves visible stage, petal differentiation and expansion stage and flowering stage) (Table 5). Full details of enriched GO and PO terms by inference from network neighbors for each of the 11 genes are given in (Figure 6 and Figure 7, and Supplementary Table S2) which also shows the networking of three classes GO terms and PO terms along with SDGs (*p* < 0.05) candidates from clusterProfiler.

## 4. Discussion

Agricultural yields are impacted by drought, an abiotic stress that is a significant environmental barrier to global agriculture. By 2025, grain yields in regions at risk of drought will need to increase by 40% to accommodate nine billion people, the projected world population by 2050 [31]. Advanced molecular breeding or biotechnological methods can be used to grow crops that are more tolerant to environmental stresses [32]. However, the identification of drought-tolerant genes and the deciphering of drought-tolerant mechanisms are the prerequisite for the application of these strategies. In this direction, lots of efforts have been made, and one of the robust approaches is a meta-analysis of large microarray datasets [33].

A wheat reference transcriptome, associated with drought, is generated for a variety of agronomically valuable crops, and the development of global transcriptomics resources for droughts in wheat is missing in research to date. Currently, the information is related to drought stress in wheat impacted by multiple kinds of geographical environments. Here, the catalog of a wheat transcriptome from multiple RNASeq collections, reported by [21,22,23,24], has provided an important resource [34] to identify mRNA’s potential roles in drought-stress conditions [35]. The analysis revealed eleven drought-responsive (11) genes, among which one (GI: 31369563, *TraesCS3D02G120200*) was common to all microarray datasets, and the other ten genes were common to three of the microarray datasets. The Ensembl Plant characterized the cluster of four genes out of them all (*TraesCS3D02G120200*, *TraesCS6D02G086600*, *TraesCSU02G154600* and *TraesCS6A02G240400*) as “Glycosyltransferase, Probable helicase MAGATAMA 3, Cytochrome c oxidase subunit 2 and D-ZIP I protein”, respectively. However, their homologous study of the remaining uncharacterized genes helped us to annotate them in other species (*Arabidopsis thaliana*, *Dichanthelium oligosanthes*, *Klebsormidium flaccidum*, *Medicago truncatula*, *Trifolium pratense*, *Utricularia gibba*, *Zea mays* and *Zoysia matrella*) and are given in Table 4. These genes have the capacity to control drought stress, so their downstream information can provide a more recent opportunity to agriculture biotechnologists.

Amongst all eleven genes, the one gene (*TraesCS3D02G120200*) found common in the four microarray datasets was stated as the standardized drought-related gene, involved in encoding glycosyltransferase. During abiotic stress conditions, secondary metabolites play an essential role in contending the stress environment because the plant’s secondary metabolism is the result of the plant’s interaction with its environment. UDP-glycosyltransferases (UGT) are enzymes that add sugars to the secondary metabolites, and thus, are believed to maintain secondary metabolites balance in plants [36]. A study revealed that UGT79B3 and UGT79B2 were involved in the glycosylation of anthocyanin and the lines with high expression of these genes assemble more anthocyanin to cope with drought stress [37]. Another study also confirmed the overexpression of glycosyltransferase in maize makes the plant drought-tolerant [38]. Overexpression of rice glycosyltransferase (UGT83A1) confers protection against salt, drought and cold stress in rice [39]. Thus, the expression level of glycosyltransferase imparts an essential part of drought tolerance in different crops.

We cannot neglect the rest of the ten genes, as they overlapped in three of the datasets used in the current study. Three (*TraesCS6D02G086600*, *TraesCSU02G154600* and *TraesCS6A02G240400*) of which contained gene descriptions in the Ensembl plant. *TraesCS6D02G086600* encodes Probable helicase MAGATAMA 3. RNA and DNA helicases plays important role in several cellular processes of protein protection and turnover. Many mechanisms involving helicases in conferring stress tolerance in plants have been put forth [40]. Overexpression of helicases alleviates abiotic stresses in chilli (*Capsicum annum* L.) [41], and *TraesCSU02G154600* encodes Cytochrome c oxidase subunit 2. In any abiotic stress, plants produce reactive oxygen species (ROS). Cytochrome c oxidase is an ideal antioxidant. None of the studies worked on cytochrome c oxidase in drought tolerance. So, this study provides a future avenue that cytochrome c oxidase might play a role in causing abiotic stress tolerance in plants. *TraesCS6A02G240400* encodes D-ZIP I protein. Specific transcription factors called homeodomain-leucine zippers (HD-Zip) are crucial in a number of developmental processes and environmental variables [42]. Different studies revealed that the overexpression of HD-Zip 1 confers tolerance to stresses [43]. All the downstream protein products including Probable helicase MAGATAMA 3; Cytochrome c oxidase subunit 2 and D-ZIP I protein are, thus, suggested to be the main reservoirs of drought response in wheat.

SBP family protein was found as a homologue of *TraesCS6D02G260700* in *Zea mays* and *Arabidopsis thaliana.* Squamous promotor-binding protein (SBP)-box genes are exclusively present in plants and encode a family member protein of transcription factors. The study showed that the overexpression of SBP family proteins imparts abiotic tolerance to different plants [44]. bHLH family protein was identified as a homologue of *TraesCS2D02G000200* in *Utricularia gibba* and Phytochrome interacting factor 3-like 6 which is one of the bHLH family proteins was found as a homologue of the same gene in *Arabidopsis thaliana* [45]. Characterize the function of a bHLH transcription factor (AhHLH112) in drought conditions. The study revealed that the AhHLH112 transcription factor resides in the nucleus and was brought on by drought stress. Moreover, the high expression of this gene ameliorates drought stress in transgenic plants in both adult and seedling stages, many studies are also in line with the same results [46]. The trihelix family protein (a DNA-binding protein), the homologue of *TraesCS6B02G234100,* has been reported to impart a role in various developmental processes of plants. Over the last several years, this gene family also takes part in abiotic stress tolerance [47]. High expression of the trihelix gene transcription factor improves drought and salt stress in different plants [48]. KNOTTED-like, from Arabidopsis thaliana 2 homologue of *TraesCS6A02G328700* in *Arabidopsis thaliana,* mechanizes processes being a DNA-binding protein as itself. The study revealed that the water-deficit stress would lead to altering the expression of this DNA-binding protein in *Pandanus amaryllifolius* [49]. AP2 (an ERF family protein) was identified as a homologue of *TraesCS5A02G456300* and *TraesCS7B02G243600* in *Zoysia matrella, Dichanthelium oligosanthes* and *Arabidopsis thaliana*. PETALA2/ETHYLENE RESPONSIVE FACTOR (AP2/ERF) family transcription factors (AP2/ERFs) are involved in abiotic stresses and respond to multiple hormones [50]. Various AP2/ERFs mutants have identified alterations in abiotic stress response and hormonal sensitivity [51]. Thus, this transcription factor may perform roles in conferring abiotic stress tolerance to plants. This demonstrates that our results could provide further new horizons as a template to make predictions about traits in crops.

To cast light on the biological interpretation of SDGs derived from meta-analysis, the selected SDGs were subjected to GO and KEGG pathway-enrichment analysis and further to plant ontology. Results revealed that the RNA polymerase II transcription factor and primary root differentiation zone annotation controlled by *TraesCS5A02G456300* and *TraesCS7B02G243600* genes were found on top of the list of enriched terms (*p* < 0.05 & Q < 0.05) revealing drought impact on plant growth. Water availability and plant growth strongly correlate as water scarcity affects cell enlargement more than cell division. This results in the inhibition of growth which ultimately led to the reduction of cell wall extensibility and turgor [52]. Results of the present study in the form of drought genes are thereby considered potential targets for getting wheat varieties showing up- or downregulation depending upon the nature of fold changes. The detailed processing of the remaining unreported genes will elucidate the consensus between the drought and molecular basis of RNA polymerase II transcription factor-dependent rooting and leaf development-related mechanisms in wheat.

## 5. Conclusions

Here, we demonstrate that the complete transcriptome-level quantitative investigation of wheat gene expression is now possible without the use of a reference genome or well-established array platforms. Comprehensive bioinformatics research revealed transcriptional pathways that linked to various genotypes and biological interventions, opening up a wealth of new possibilities for fundamental researchers and wheat breeders. The online repository, such as NCBI, is highly helpful for the construction of the predicted standard drought genes across the world, in addition to applications for resolving fundamental biological concerns, as stated in this work. We think that our strategy is broadly applicable to drought stress and will be perfect for the research of important agronomic qualities that will, undoubtedly, be influenced by the food security agenda in the future. This work will become more widely available as it has been shown to produce reliable reference drought gene groups. In fact, it might end up serving as a systems-based approach’s main workhorse for drought response in wheat with scarce public genetic resources. This manuscript specifically establishes the groundwork for a precise molecular-level examination of root growth processes for the wheat crop.

## Figures and Tables

**Table 1:** The SDGs were screened based on 1-sample proportions test with Bonferroni corrected *p* < 0.05, discarding other genes that did not become significant with similar criteria. The differential expression data included 4859, 12,723, 172 and 2091 genes from GSE45563, GSE47090, GSE70443 and GSE87325 microarray experiments, respectively.

Sr #	GSE45563	GSE47090	GSE70443	GSE87325	Coutif	*p*-Value
1	31369563	31369563	31369563	31369563	4	4.16 × 10^−23^
2	25245708	25245708	25245708	#N/A	3	9.58 × 10^−7^
3	31369545	31369545	31369545	#N/A	3	9.58 × 10^−7^
4	25232126	25232126	25232126	#N/A	3	9.58 × 10^−7^
5	#N/A	19959095	19959095	19959095	3	9.58 × 10^−7^
6	14320	14320	14320	#N/A	3	9.58 × 10^−7^
7	25550165	25550165	25550165	#N/A	3	9.58 × 10^−7^
8	25270582	25270582	25270582	#N/A	3	9.58 × 10^−7^
9	20313737	20313737	20313737	#N/A	3	9.58 × 10^−7^
10	20334251	20334251	20334251	#N/A	3	9.58 × 10^−7^
11	25231635	25231635	25231635	#N/A	3	9.58 × 10^−7^

**Table 2:** Gene ID conversion provided the information about SDGs in the form of columns. First and second columns are the IDs that resulted from differential expression analyses. Similarly, third column was filled with their respective IWGSC IDs due to their high numbers in the form of alignment scores and residual identity in percentages with low E-values. Genomic locations were recorded by searching ENSEMBL database and provided as last three columns.

Genes	Accession Numbers	IWGSC Genes	Score	E-Value	Identity (%)	Chromosome	Start	End
31369563	CD454935.1	*TraesCS3D02G120200*	484	0	97.9	3D	75946541	75948546
25245708	CA667105.1	*TraesCS6A02G328700*	146	1.50 × 10^−75^	99.3	6A	562271850	562272738
31369545	CD454917	*TraesCS2D02G000200*	424	0	99.5	2D	39478	40878
25232126	CA653601	*TraesCS6D02G086600*	248	2.50 × 10^−136^	100	6D	52285210	52292603
19959095	BJ220896	*TraesCS6D02G260700*	696	0	99.9	6D	368305109	368306633
14320	X52867	*TraesCSU02G154600*	507	0	98.3	Un	206782100	206784099
25550165	CA734567	*TraesCS6B02G234100*	105	9.40 × 10^−51^	100	6B	393072749	393090605
25270582	CA684029	*TraesCS1D02G333000*	118	1.30 × 10^−58^	96.2	1D	423261565	423269992
20313737	BQ168410	*TraesCS5A02G456300*	224	6.00 × 10^−122^	100	5A	636429039	636430690
20334251	BQ172428	*TraesCS6A02G240400*	308	5.40 × 10^−172^	99.4	6A	451659872	451662196
25231635	CA653110	*TraesCS7B02G243600*	235	3.20 × 10^−128^	99.6	7B	452150743	452210823

**Table 3:** SDGs ontology created using the reference annotation of IWGSC wheat genomes. For this purpose, drought protein sequences were annotated using R v3.4.1. Majority of genes ontology annotations were predicted for wheat sequences as given below.

GO Type	Description	Negative Log10 (*p* Adjusted)	ID	Gene Ratio	Bg. Ratio	*p* Value	P Adjust	Q Value	Gene ID	Count
BP	regulation of autophagy	2.107492695	GO:0010506	¼	3/7682	0.001561	0.007807	0.001644	25270582	1
	response to auxin	1.683984604	GO:0009733	¼	25/7682	0.012957	0.020702	0.004358	25245708	1
	electron transport chain	1.683984604	GO:0022900	¼	31/7682	0.016047	0.020702	0.004358	14320	1
	regulation of transcription from RNA polymerase II promoter	1.683984604	GO:0006357	¼	32/7682	0.016562	0.020702	0.004358	20334251	1
CC	Mon1-Ccz1 complex	3.155214539	GO:0035658	1/5	1/7148	0.000699	0.000699	#N/A	25270582	1
MF	RNA polymerase II transcription factor activity, sequence-specific DNA binding	2.290980192	GO:0000981	¼	2/7814	0.001024	0.005117	0.001616	20334251	1
	D-threo-aldose 1-dehydrogenase activity	2.290980192	GO:0047834	¼	2/7814	0.001024	0.005117	0.001616	31369545	1
	alditol:NADP+ 1-oxidoreductase activity	2.290980192	GO:0004032	¼	3/7814	0.001535	0.005117	0.001616	31369545	1
	cytochrome-c oxidase activity	2.048275717	GO:0004129	¼	7/7814	0.003579	0.008948	0.002826	14320	1

**Table 4:** Searching the homologs of SDGs in annotated genomes of Arabidopsis thaliana, Dichanthelium oligosanthes, Klebsormidium flaccidum, Medicago truncatula, Trifolium pratense, Utricularia gibba, Zea mays and Zoysia matrella.

Drought Genes	Hit ID	Organism	Description	Score	E-Value
*TraesCS6D02G260700*	Zmw_sc01257.1. g00130.1	*Zea mays*	*Zoysia matrella* SBP family protein	110	7.00 × 10^−6^
*TraesCS6D02G260700*	AT1G53160.1	*Arabidopsis thaliana*	*Arabidopsis thaliana* squamosa promoter-binding protein-like 4	278	3.00 × 10^−29^
*TraesCS6A02G240400*	AT2G46680.2	*Arabidopsis thaliana*	*Arabidopsis thaliana* homeobox 7	264	6.00 × 10^−27^
*TraesCS2D02G000200*	678321642	*Utricularia gibba*	*Utricularia gibba* bHLH family protein	138	3.00 × 10^−8^
*TraesCS2D02G000200*	AT3G59060.4	*Arabidopsis thaliana*	phytochrome interacting factor 3-like 6	-	3.00 × 10^−18^
*TraesCS6D02G086600*	kfl00432_0070	*Klebsormidium flaccidum*	*Klebsormidium flaccidum* C3H family protein	709	2.00 × 10^−78^
*TraesCS6D02G086600*	AT1G29560.2	*Arabidopsis thaliana*	*Arabidopsis thaliana* C3H family protein	101	1.00 × 10^−4^
*TraesCS6B02G234100*	Medtr2g092960.1	*Medicago truncatula*	*Medicago truncatula* Trihelix family protein	104	0.001
*TraesCS6B02G234100*	AT3G58630.1	*Arabidopsis thaliana*	sequence-specific DNA binding transcription factors	-	2.00 × 10^−24^
*TraesCS6A02G328700*	AT1G70510.1	*Arabidopsis thaliana*	*Arabidopsis thaliana* KNOTTED-like from Arabidopsis thaliana 2	64	0.04
*TraesCS1D02G333000*	No hits found				
*TraesCS5A02G456300*	Zmw_sc03344.1. g00030.1	*Zoysia matrella*	*Zoysia matrella* ERF family protein	160	1.00 × 10^−11^
*TraesCS5A02G456300*	AT5G19790.1	*Arabidopsis thaliana*	*Arabidopsis thaliana* related to AP2 11	229	4.00 × 10^−22^
*TraesCS3D02G120200*	Tp57577_TGAC_v2_mRNA33215	*Trifolium pratense*	*Trifolium pratense* bHLH family protein	325	5.00 × 10^−31^
*TraesCS3D02G120200*	AT2G22760.1	*Arabidopsis thaliana*	bHLH family protein	-	1.00 × 10^−20^
*TraesCS7B02G243600*	Do013987.1	*Dichanthelium oligosanthes*	*Dichanthelium oligosanthes* ERF family protein	244	1.00 × 10^−20^
*TraesCS7B02G243600*	AT5G19790.1	*Arabidopsis thaliana*	related to AP2 11	-	2.00 × 10^−18^

**Table 5:** Plant ontology created using the reference annotation of *Arabidopsis thaliana* and *Zea mays* genomes. For this purpose, drought protein sequences were checked for homology across the available annotated sequences. Majority of homologs were found and plant ontology annotations were assigned to wheat sequences as given below:.

ID	Description	Gene Ratio	Bg Ratio	*p* Value	P Adjust	Q Value	Gene ID	Count
PO:0003015	primary root differentiation zone	2/9	42/24102	0.000106	0.001957	0.000309	*TraesCS5A02G456300/TraesCS7B02G243600*	2
PO:0005679	Epidermis	2/9	57/24102	0.000196	0.001957	0.000309	*TraesCS5A02G456300/TraesCS7B02G243600*	2
PO:0000256	root hair cell	2/9	208/24102	0.002564	0.015719	0.002482	*TraesCS5A02G456300/TraesCS7B02G243600*	2
PO:0004707	fruit dehiscence zone	1/9	11/24102	0.004101	0.015719	0.002482	*TraesCS3D02G120200*	1
PO:0020128	leaf margin	1/9	16/24102	0.00596	0.015719	0.002482	*TraesCS3D02G120200*	1
PO:0009064	receptacle	1/9	17/24102	0.006331	0.015719	0.002482	*TraesCS6A02G328700*	1
PO:0000112	shoot axis epidermis	1/9	18/24102	0.006702	0.015719	0.002482	*TraesCS3D02G120200*	1
PO:0000033	fruit valve	1/9	19/24102	0.007074	0.015719	0.002482	*TraesCS3D02G120200*	1
PO:0004006	mesophyll cell	1/9	19/24102	0.007074	0.015719	0.002482	*TraesCS3D02G120200*	1
PO:0006016	leaf epidermis	1/9	23/24102	0.008557	0.017114	0.002702	*TraesCS3D02G120200*	1
PO:0009053	Peduncle	1/9	31/24102	0.011518	0.019813	0.003128	*TraesCS3D02G120200*	1
PO:0020127	primary root	1/9	32/24102	0.011888	0.019813	0.003128	*TraesCS3D02G120200*	1
PO:0006036	root epidermis	1/9	62/24102	0.022919	0.035259	0.005567	*TraesCS3D02G120200*	1
PO:0006504	leaf trichome	1/9	81/24102	0.029848	0.04264	0.006733	*TraesCS3D02G120200*	1

## Data Availability

All the data is presented in the main text and Supplementary File.

## Supplementary Materials

The following supporting information can be downloaded at: https://www.mdpi.com/article/10.3390/genes13101906/s1, Table S1: Details of transcriptome data (GSE87325, GSE47090, GSE45563 and GSE70443), Table S2: Details related to GO and PO.
